# Characterization of mRNA and microRNA in human mast cell-derived exosomes and their transfer to other mast cells and blood CD34 progenitor cells

**DOI:** 10.3402/jev.v1i0.18389

**Published:** 2012-04-16

**Authors:** Karin Ekström, Hadi Valadi, Margareta Sjöstrand, Carina Malmhäll, Apostolos Bossios, Maria Eldh, Jan Lötvall

**Affiliations:** 1 Krefting Research Centre, Sahlgrenska Academy, University of Gothenburg, Gothenburg, Sweden; 2 Department of Biomaterials, Sahlgrenska Academy, University of Gothenburg, Gothenburg, Sweden; 3 BIOMATCELL VINN Excellence Center of Biomaterials and Cell Therapy, Gothenburg, Sweden; 4 Department of Rheumatology and Inflammation Research, Sahlgrenska Academy, University of Gothenburg, Gothenburg, Sweden

**Keywords:** exosomes, extracellular vesicles, human mast cells, RNA, esRNA

## Abstract

**Background:**

Exosomes are nanosized vesicles of endocytic origin that are released into the extracellular environment by many different cells. It has been shown that exosomes from various cellular origins contain a substantial amount of RNA (mainly mRNA and microRNA). More importantly, exosomes are capable of delivering their RNA content to target cells, which is a novel way of cell-to-cell communication. The aim of this study was to evaluate whether exosomal shuttle RNA could play a role in the communication between human mast cells and between human mast cells and human CD34^+^ progenitor cells.

**Methods:**

The mRNA and microRNA content of exosomes from a human mast cell line, HMC-1, was analysed by using microarray technology. Co-culture experiments followed by flow cytometry analysis and confocal microscopy as well as radioactive labeling experiments were performed to examine the uptake of these exosomes and the shuttle of the RNA to other mast cells and CD34^+^ progenitor cells.

**Results:**

In this study, we show that human mast cells release RNA-containing exosomes, with the capacity to shuttle RNA between cells. Interestingly, by using microRNA microarray analysis, 116 microRNAs could be identified in the exosomes and 134 microRNAs in the donor mast cells. Furthermore, DNA microarray experiments revealed the presence of approximately 1800 mRNAs in the exosomes, which represent 15% of the donor cell mRNA content. In addition, transfer experiments revealed that exosomes can shuttle RNA between human mast cells and to CD34^+^ hematopoietic progenitor cells.

**Conclusion:**

These findings suggest that exosomal shuttle RNA (esRNA) can play a role in the communication between cells, including mast cells and CD34^+^ progenitor cells, implying a role in cells maturation process.

Exosomes are 30–100 nm membrane vesicles that are secreted into the extracellular environment by many different cell types. These small extracellular vesicles are formed by inward budding of late endosomes and are released into the extracellular environment upon fusion with the plasma membrane (1). Exosomes are released by a wide range of cells, including mast cells (MC) (2), dendritic cells (DC) (3), tumour cells (4), reticulocytes (5), epithelial cells (6) and B-cells (7). Exosomes have also been found in a number of human body fluids, including blood plasma (8), urine (9), breast milk (10), amniotic fluid (11), malignant ascites (12) and bronchoalveolar lavage fluid (13), indicating importance in vivo.

In 1996, it was discovered that exosomes have an immunological function and consequently from then the immunological role of exosomes has been studied extensively (7). Exosomes have been shown to take part in both T cell activation (14) and in tolerance development (15). It has been shown that exosomes released from mast cells have the capacity to activate T cells (16) and endothelial cells (17), and in addition to induce DC maturation (18). Thus, there is now extensive evidence that exosomes can mediate communication between cells over a distance. Furthermore, exosomes primed with specific tumour antigens are under clinical trials for cancer treatment (19).

In 2007, we showed that exosomes from mast cells contain both mRNA and microRNA and that these RNAs can be transferred to other mast cells. In addition, we showed that the exosomal RNA was functional in the recipient cells by translation of exosomal mRNA into proteins in the recipient cells (20). Subsequently, many other studies have shown that exosomes can shuttle RNA between cells and, in that way, modify the recipient cells both by translation of the exosomal mRNA into proteins and by repressing the translation in the case of microRNAs (20–26). The function of exosomes depends on the cellular origin as well as the condition for the producing cells, which give the exosomes their characteristic composition (1, 27). For example, exosomes originating from cells exposed to oxidative stress convey protective messages against stress in the recipient cells (27). Together, these findings suggest that the exosomal shuttle of RNA can change the biological function of the recipient cell.

The detailed RNA content of exosomes from human mast cells has so far not been determined. In this study, we have characterized the mRNA and microRNA content of exosomes from a human mast cell line, HMC-1, by using microarray technology. We have also determined the hypothetical function of these RNAs using Ingenuity Pathway Analysis (IPA). Furthermore, we show that exosomal shuttle of RNA occur between human mast cells and human hematopoietic CD34^+^ progenitor cells.

## Results and discussion

### Exosome characterization

**Fig. 1:**
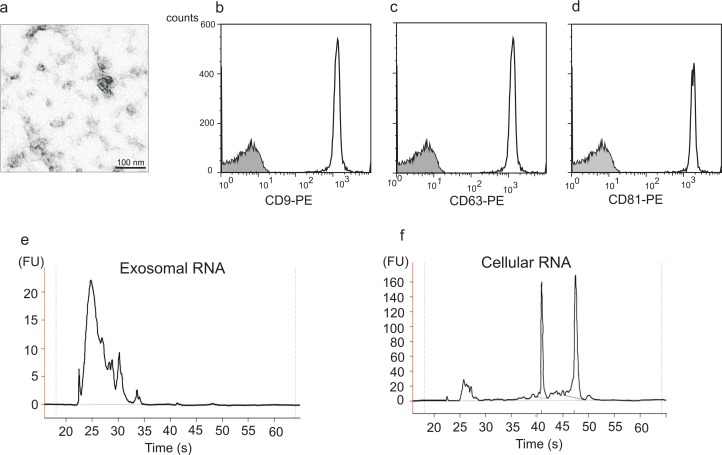
Human mast cells produce exosomes that contain RNA. (a) Transmission electron microscopy picture of exosomes isolated from the supernatant of the human mast cell line HMC-1 by a method based on repeated centrifugation and filtration steps followed by high speed ultracentrifugation. Exosomes are visible as small 40–80 nm vesicles, scale bar 100 nm. (b-d) Flow cytometry detection and characterization of exosomes captured onto anti-CD63 coated beads. The exosome-bead complexes were immunostained against the tetraspanins (open curve) CD9 (b), CD63 (c) and CD81 (d) and compared with the appropriate isotype control (filled curve) (e, f) Bioanalyzer^®^ results of total RNA isolated from exosomes (e) and their donor HMC-1 cells (f) shows the size distribution of RNA. The two dominant peaks in cellular RNA correspond to 18S and 28S rRNA, respectively. These ribosomal peaks are not dominant in the exosomal RNA (FU, fluorescence units).

Exosomes released from the human mast cell line HMC-1 were isolated from the cell supernatant through a series of filtration and centrifugation steps. Exosomes were visualized by electron microscopy as small vesicles, typically 40–80 nm in diameter (Fig. 1a). Flow cytometric analysis of exosomes attached to anti-CD63 latex beads revealed that they were positive for the tetraspanins CD9, CD63 and CD81, which are proteins often enriched in the exosomal membrane [Fig. 1b–1d; (28)]. Since there are no specific markers for exosomes, different methods are often combined for the detection of exosomes, and the combination of electron microscopy and flow cytometry is very much in line with what other investigators use (8, 29). These data therefore confirm that the studied extracellular vesicles indeed are exosomes. The RNA content of exosomes and their donor cells was examined using capillary electrophoresis (Fig. 1e and 1f). This confirmed that HMC-1 exosomes contain RNA and that the RNA pattern of exosomes and cells differs, where the exosomes lack the dominant ribosomal RNA peaks. The finding that human mast cell-derived exosomes contain RNA is in line with our previous original finding, which showed that mouse mast cell-derived exosomes contain RNA. This has been further confirmed as exosomes from various origins, including glioblastoma cells, airway epithelial cells, human saliva, human urine and human plasma, have also been shown to contain RNA (20–22, 30–32). Furthermore, it is known that the exosomal RNA is functional, as we and others have shown that the mRNA found in several different types of exosomes can translate to proteins (20, 21).

### Exosomal mRNA profile

**Fig. 2:**
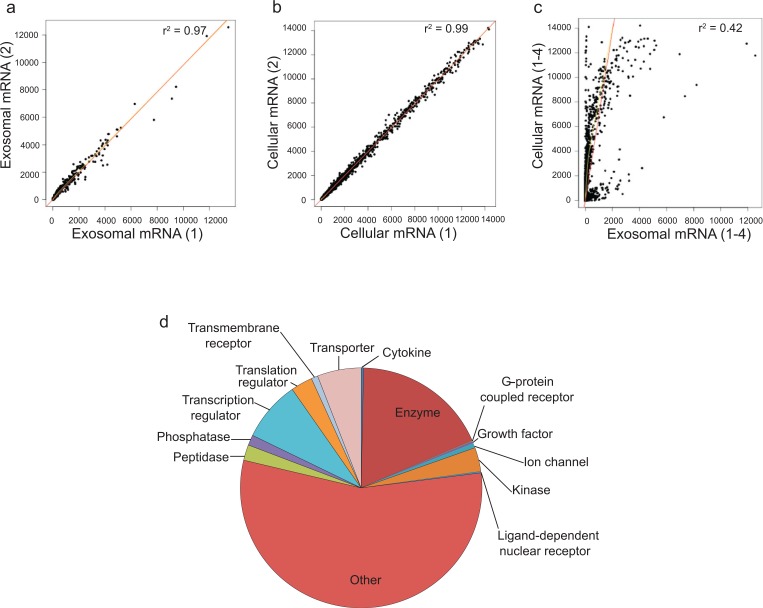
Characterization of exosomal mRNA. Affymetrix DNA microarray was applied to identify mRNA from exosomes and their donor HMC-1 cells (n=4). (a,b) Correlation of gene expression signals within exosomes and cells samples are shown. In total, 1,849 mRNAs could be found in exosomes and 12,346 in the cells. (c) There was no strong correlation between the gene expression signals in exosomes and cells. (d) Relative group functions of the exosomal mRNA according to Ingenuity Software Analysis.

The mRNA content of HMC-1 exosomes and their donor cells was analysed using Affymetrix DNA microarray (n =4, Fig. 2a–2d). This revealed the presence of ~1,800 transcripts in the HMC-1 exosomes (Fig. 2a, Additional Information, Table S1). The number of detected mRNA in the exosomes was 15% of the mRNA detected in the donor HMC-1 cells (~12,300 mRNA transcripts, Fig. 2b, Additional Information, Table S2). Fifty-four mRNAs were expressed in exosomes but were not detected in the HMC-1 cells according to the analysis cutoff settings (Additional Information, Table S3). Interestingly, there was a clear expression difference in the mRNA profile between cells and exosomes (Fig. 2c). The relative group functions of the exosomal mRNA according to IPA are illustrated in Figure 2d.

The presence of mRNA in exosomes derived from human mast cells suggests that exosomes can transfer genetic signals from one cell to another and result in protein production in the recipient cell. This functionality of the esRNA have been documented in previous studies, using both mouse mast cells as well as glioblastoma cell cultures in vitro (20, 21). Therefore, it is not unreasonable to hypothesize that the transfer of specific mRNA, from one cell to another, via exosomes is a very powerful tool for a cell to deliver messages to other cells, as the recipient cell would produce numerous proteins from each mRNA that is delivered. Theoretically, this would be more efficient than cell-to-cell signalling with proteins per se. Interestingly, there was a clear difference in the mRNA profile of cells and exosomes (Fig. 2c). Thus, it is unlikely that a bulk part of the mRNA in exosomes is a random sample of the cellular mRNA but rather specifically packed into the exosomes by a specific mechanism.

### Exosomal microRNA profile

**Fig. 3:**
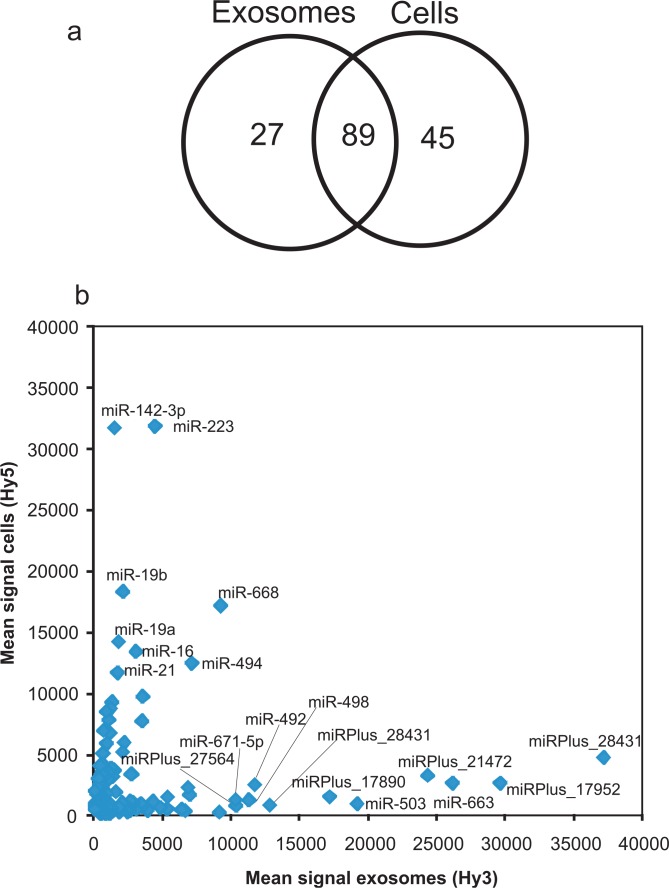
Characterization of exosomal microRNA. miRCURY™ LNA array analysis was applied to detect microRNAs in exosomes (Hy3) and their donor HMC-1 cells (Hy5) (n=3). (a) In total, 116 microRNAs were detected in exosomes and 134 microRNAs in their donor HMC-1 cells. Twenty-seven microRNAs were only detected in exosomes, 45 were only detected in cells and 89 were found in both cells and exosomes. (b) Scatterplot showing the mean intensity signals for microRNAs in exosomes (Hy3) and cells (Hy5). A number of microRNAs were found to be differently expressed in exosomes compared to their donor cells. The annotations for microRNAs with mean signals>10,000 are indicated in the plot. miRPLus_are sequences not yet included in miRBase.

Since exosomes derived from mouse MC/9 mast cells have been shown to contain microRNA (20), together with that the RNA profiling revealed the presence of small RNA in the HMC-1 exosomes, we hypothesized that at least a fraction of the small RNA indeed is represented by microRNA. The presence and levels of specific microRNAs from both exosomes and their donor cells was determined using miRCURY™ LNA array analysis. In the exosomes, 116 microRNAs were identified and 134 microRNAs were identified in the donor HMC-1 cells (Fig. 3a and 3b, Table I and Additional Information, Table S4). Some of these were more abundant in exosomes than in the donor cells, and 27 microRNAs were found only in the exosomes. The 10 most abundant microRNAs in the exosomes, compared to the cells, were hsa-miR-451, hsa-miR-503, miRPlus_27560, miRPlus_2843, miRPlus_27564, hsa-miR-583, miRPlus_1795, miRPlus_17890, hsa-miR-663 and hsa-miR-30b. Thirty-two microRNAs were found to be at least 4-fold increased in exosomes compared to the donor cells, and 41 microRNAs were at least 4-fold decreased, which was highly consistent between the three different experiments. This implies a sorting mechanism also for microRNAs into the exosomes from the donor cells, suggesting that neither the mRNA or the microRNA content in exosomes is a random sample of the cellular RNA pool. In contrast, it has been shown that the microRNA profile of exosomes derived from tumour cells correlates with the microRNA expression profiles of the originating tumour cells, implying a use for exosomes as biomarkers (33).

**Table I:** MicroRNA profiling in exosomes and donor HMC-1 cells

Elevated in exosomes (≥ log2 fold change)	Equal in exosomes and cells (>−log2 <log2)	Elevated in cells (≤−log 2 fold change)
hsa-miR-451, hsa-miR-503, miRPlus_27560, miRPlus_28431, miRPlus_27564, hsa-miR-583, miRPlus_17952, miRPlus_17890, hsa-miR-663, hsa-miR-30b*, hsa-miR-498, miRPlus_17900, hsa-miR-671-5p, miRPlus_27561, hsa-miR-125b-1*, miRPlus_21472, hsa-miR-765, miRPlus_28993, hsa-miR-122, hsa-miR-483-5p, hsa-miR-296-5p, hsa-miR-96*, hsa-miR-628-3p, hsa-miR-193a-5p, hsa-miR-518c*, hsa-miR-637, hsa-miR-492, hsa-miR-371-5p, hsa-miR-198, hsa-miR-658, hsa-miR-210	miRPlus_17921, hsa-miR-423-5p, hsa-miR-611, hsa-miR-623, miRPlus_17832, hsa-miR-557, hsa-miR-183*, hsa-miR-630, miRPlus_17869, hsa-miR-516b, miRPlus_28575, hsa-miR-185, hsa-miR-617, hsa-miR-518e*/519a*/519b-5p/519c-5p/522*/523*, hsa-miR-602, hsa-miR-129-5p, miRPlus_28232, hsa-miR-184, hsa-miR-665, hsa-miR-518a-5p/527, miRPlus_17896, hsa-miR-518d-5p/518f*/520c-5p/526a, hsa-miR-501-3p, hsa-miR-193b, hsa-miR-22, hsa-miR-766, hsa-miR-483-3p, hsa-miR-148b, hsa-miR-512-5p, hsa-miR-519e*, hsa-miR-185*, hsa-miR-34a, hsa-miR-500*, hsa-miR-502-3p, hsa-miR-801, hsa-miR-24, hsa-miR-130b, hsa-miR-148a, hsa-miR-99b, hsa-let-7d, hsa-miR-92b, hsa-miR-365, miRPlus_17945, hsa-miR-494, hsa-miR-107, hsa-miR-668, miRPlus_17865, hsa-miR-378, hsa-miR-422a_MM2, hsa-miR-92a, hsa-miR-221, hsa-miR-574-5p, miRPlus_17861, hsa-miR-125a-5p, hsa-miR-29a, hsa-miR-15a, hsa-miR-15a, hsa-miR-32*, hsa-miR-9*, miRPlus_27839, hsa-miR-320, hsa-miR-23b, hsa-miR-29c, hsa-miR-146a, hsa-miR-10a, hsa-miR-23a,hsa-miR-151-5p,hsa-miR-15b, hsa-miR-186, hsa-miR-30e*, hsa-miR-338-3p,hsa-miR-361-5p, hsa-miR-342-3p, hsa-miR-222,hsa-miR-191, miRPlus_11201, hsa-miR-30d, hsa-miR-29b, hsa-miR-193a-3p, hsa-miR-126, hsa-miR-20b*, hsa-miR-27b, hsa-let-7c, hsa-let-7i, hsa-miR-744, hsa-miR-425, hsa-miR-340, hsa-let-7b	hsa-miR-93, hsa-miR-16, hsa-miR-491-3p, hsa-miR-9, hsa-miR-103, hsa-miR-374a, hsa-miR-27a, hsa-miR-98, hsa-miR-934, hsa-miR-590-5p, hsa-miR-487b, hsa-miR-30a, hsa-miR-30c, hsa-miR-374b,hsa-let-7f, hsa-miR-195, hsa-miR-301a, hsa-miR-26a_MM1, hsa-miR-30e, hsa-miR-26a, hsa-miR-146b-5p, hsa-miR-20b, hsa-miR-519d, hsa-miR-106b, hsa-miR-21, hsa-miR-768-5p, hsa-miR-20a, hsa-miR-17, hsa-miR-106a, hsa-miR-223, hsa-miR-30b, hsa-miR-101, hsa-miR-18b, hsa-miR-19a, hsa-miR-18a, hsa-miR-19b, hsa-miR-212, hsa-miR-142-5p, hsa-let-7a, hsa-miR-32, hsa-miR-768-3p, hsa-miR-142-3p
Exosomal microRNA was labelled with Hy3 and cellular microRNA with Hy5 and analysed using miRCURY™ LNA array, version 9.2. Each signal was normalised against the background and with global Lowess regression algorithm. Fold change (exosomes/cells) was calculated and the data were log2 transformed. The microRNAs were divided into 3 groups [elevated in exosomes (≥log2 fold change), Equal between exosomes and cells (>−log2 <log2) or elevated in cells (≤−log 2 fold change)] depending on their log2 values. For more details, see the Method section and Additional Information Table S4.		

In theory, the exosomal microRNAs have the capacity to regulate the translation of many different mRNAs in the recipient cell, illustrating the extensive regulatory capacity of exosomal microRNAs (34). Furthermore, many small regulatory RNAs are being developed for therapeutic use in human disease, but it is not yet clear how they can be delivered in the most efficient way. Exosomes may be an ideal vector for microRNA delivery to cells, as they are endogenous and shown to be able to deliver RNA to recipient cells both in vitro and in vivo (8, 20, 21, 35).

### Exosome mRNA functions, pathways and networks

**Fig. 4:**
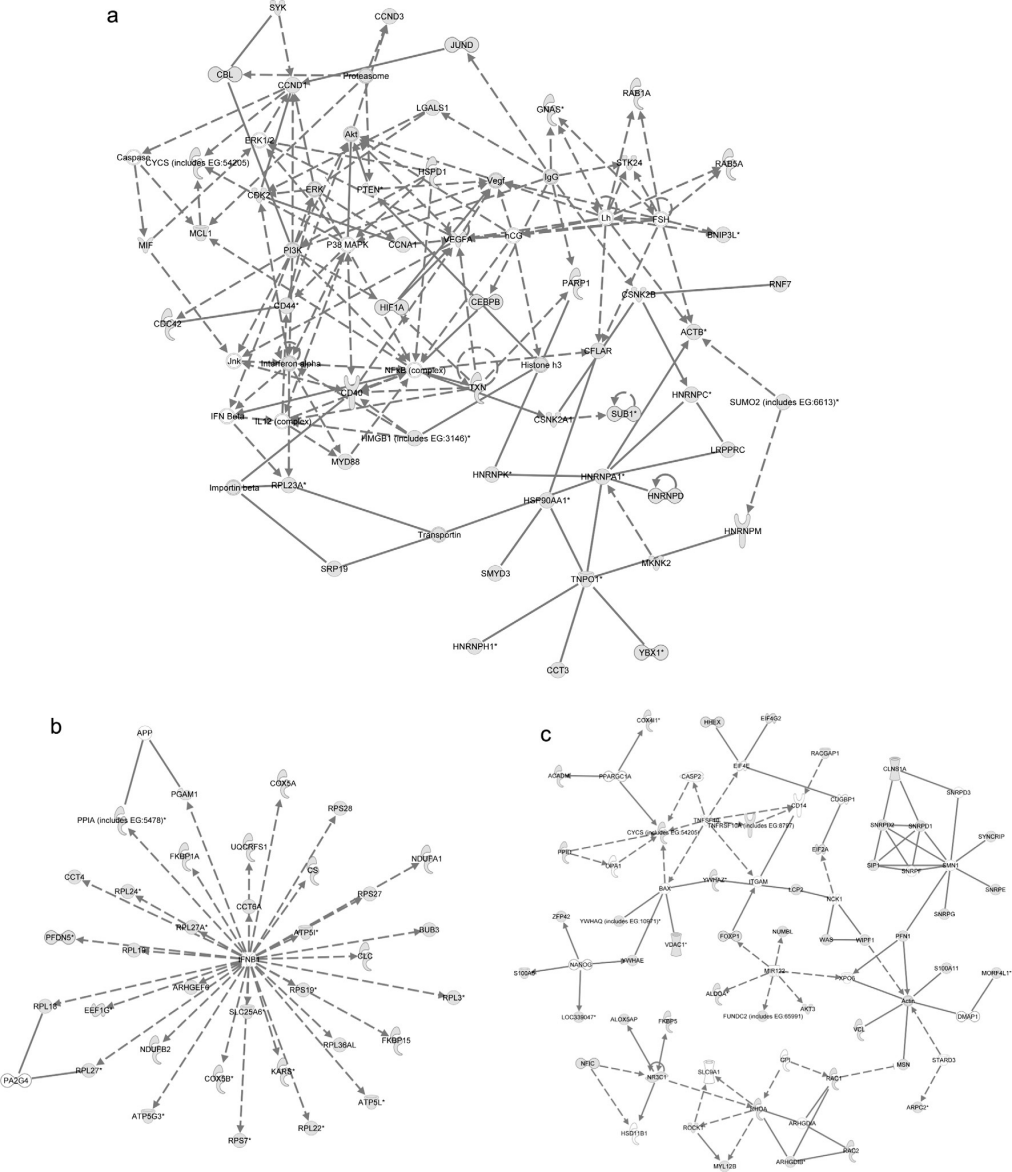
Network-based analysis of exosomal mRNA. IPA was applied to predict networks for the exosomal mRNA identified by Affymetrix DNA microarray. The top three predicted networks for exosomal mRNA were (a) “cellular development, hematological system development and function, hematopoiesis” (50 focus genes, score 34), (b) “protein synthesis, post-translational modification, protein folding” (34 focus genes, score 30) and (c) “cell death, RNA post-transcriptional modification, cellular assembly and organization” (47 focus genes, score 30). Continuous lines indicate direct interaction and dotted lines indirect interaction. Focus genes are labelled in grey and genes not included in the dataset are white. For more information please see the IPA web site (www.ingenuity.com/).

Using IPA, we investigated the functions, canonical pathways and networks for the exosomal mRNA from the Affymetrix microarray analysis. The 3 networks with the highest score were “cellular development, hematological system development and function and hematopoiesis” (score 34; Fig. 4a), “protein synthesis, post-translational modification and protein folding” (score 30; Fig. 4b) and “cell death, RNA post-transcriptional modification and cellular assembly and organization” (score 30; Fig. 4c). The top 5 canonical pathways (previously established networks) that were identified are “oxidative phosphorylation,” “mitochondrial dysfunction,” “EIF2 signalling,” “regulation of actin-based mobility by Rho” and “protein ubiquitination pathway.” The top biological functions of the exosomal mRNAs, according to IPA, are listed in Additional Information, Table S5. Interestingly, the top cellular and molecular functions for the exosomal mRNA are protein synthesis, cell death, RNA-post transcriptional modification, cellular growth and proliferation and post-transcriptional modification. Interestingly, the predicted networks and functions for HMC-1 exosomes are in line with the previously identified network for the mouse mast cell exosomes (cellular development, protein synthesis and RNA post-transcriptional modification) (20).

### Exosome microRNA network analysis

**Fig. 5:**
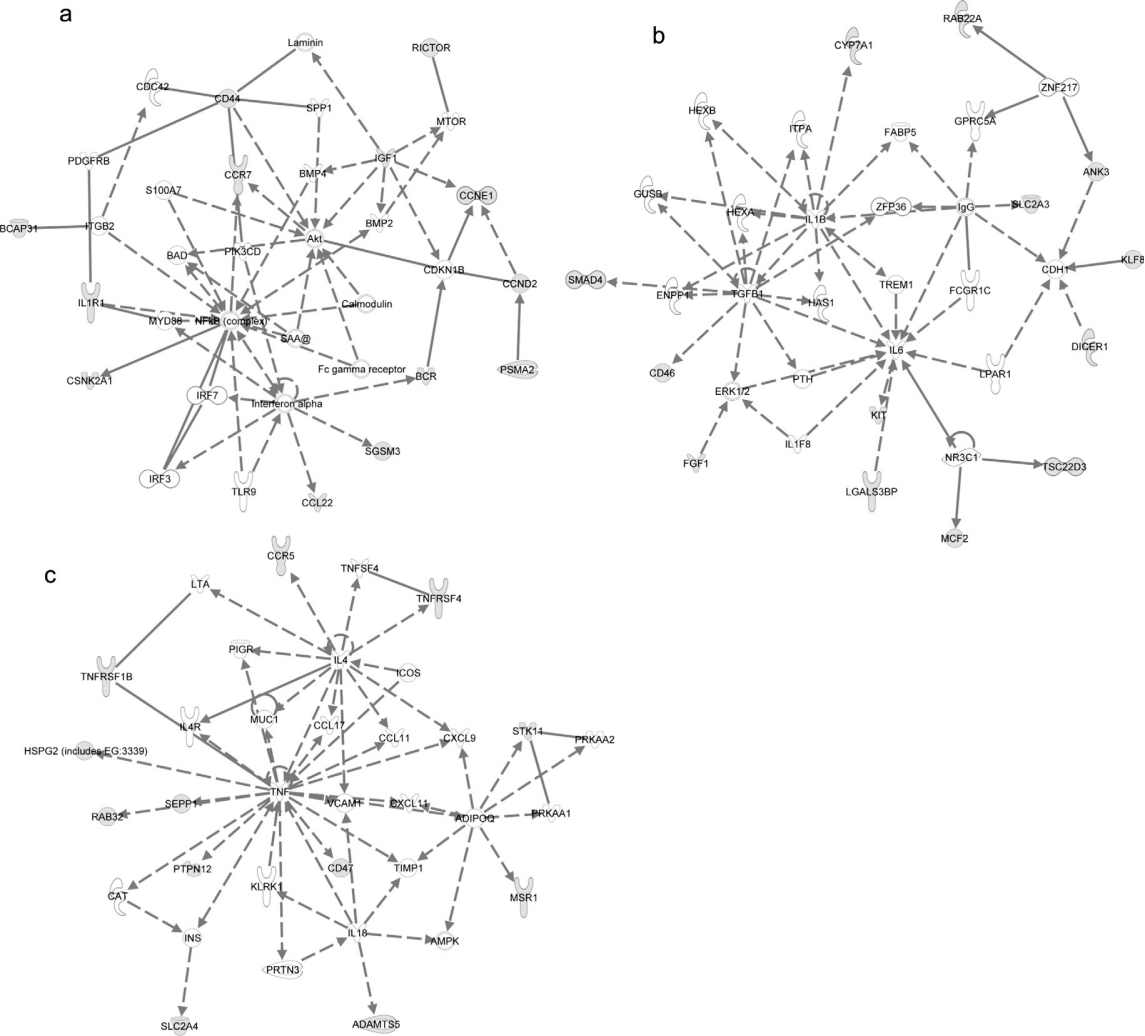
Networks predicted to be regulated by exosomal microRNA. Predicted targets for the top 5 annotated miRNA in exosomes were searched using TargetScan to predict target mRNA and subsequently analysed by IPA software to determine predicted networks regulated by exosomal microRNA. The top 3 predicted networks regulated by exosomal microRNA were (a) “cellular development, cellular movement, hematological system development and function” (13 focus molecules, score 12), (b) “cellular function and maintenance, cellular development, neurological disease” (13 focus molecules, score 12) and (c) “cellular movement, hematological system development and function, immune cell trafficking” (12 focus molecules, score 11).

Predicted targets for the top 5 annotated miRNA in exosomes (miR-663, miR-503, miR-492, miR-498 and miR-671–5p) were searched using TargetScan and subsequently the target mRNAs were analysed by IPA software to determine predicted canonical pathways, networks and biofunctions regulated by the exosomal miRNAs (Fig. 5 and Additional Information, Table S6.). The top 3 networks that were predicted to be regulated by exosomal miRNA were “cellular development, cellular movement, hematological system development and function” (score 12, Fig. 5a), “cellular function and maintenance, cellular development, neurological disease” (score 12, Fig. 5b) and “cellular movement, hematological system development and function, immune cell trafficking” (score 11, Fig. 5c). The top 5 canonical pathways predicted to be affected by the top five microRNAs were “cell cycle: G1/S checkpoint regulation,” “glycosphingolipid biosynthesis – lactoseries,” “glycosphingolipid biosynthesis – neolactoseries,” “methionine metabolism” and “hepatic fibrosis/hepatic stellate cell activation.”

### Uptake of HMC-1 exosomes by different cells

**Fig. 6:**
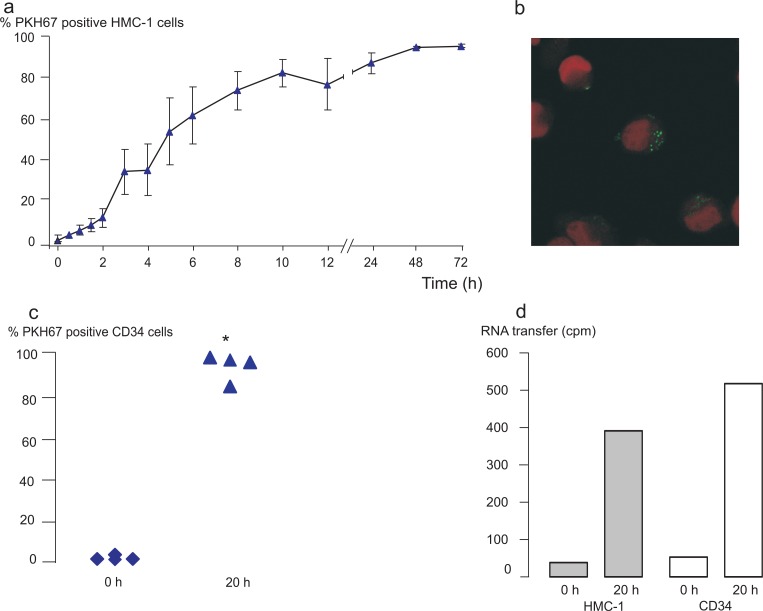
Exosomes can transfer RNA to HMC-1 cells and CD34 positive progenitor cells. PKH67-labelled (green) exosomes were added to HMC-1 or CD34 cells in culture. Cells were harvested, washed and analysed in the flow cytometer or by confocal microscopy at different time points. (a) Shows the percentage of HMC-1 cells positive for PKH67 after the different time points. (b) Confocal microscopy picture of green exosomes internalized by HMC-1 cells (red nuclei stained with 7-AAD). (c) CD34 positive cells take up PKH67-labelled exosomes after 20 hours compared to 0 hour. (d) Exosomes were radioactively labelled, by culturing HMC-1 donor cells in medium supplemented with ^3^H-uridine. Exosomes were isolated, washed and added to HMC-1 and CD34 cells in culture. Cells were harvested after 0 hour and 20 hours, washed and the radioactive signal quantified. The data show that exosomal RNA is shuttled to HMC-1 (n=2) and CD34 (n=1) cells. Values in (a) and (c) are mean ± SEM, n=4 and in C, *p<0.05.

Since the mRNA and microRNA network analyses suggested a role for the exosomal mRNA in hematopoiesis and cellular development, we hypothesized that exosomes can be taken up by and influence not only other mast cells but also CD34^+^ hematopoietic progenitor cells. To determine this, exosomes were labelled with the green fluorescent membrane dye PKH67 and added to HMC-1 cells and CD34^+^ hematopoietic progenitor cells obtained from human blood. The results from flow cytometry analysis revealed that both HMC-1 cells and CD34^+^ cells were able to take up the PKH67-labelling from the exosomes, indicating that the exosomes indeed are taken up by the recipient cells (Fig. 6a and 6c). After 1 hour, 10 ± 2% of the HMC-1 cells were positive for PKH67. Within 4 hours, 41 ± 13% and within 8 hours, 79 ± 9% of the cells were positive for PKH67 (Fig. 6a, n=4). The uptake of exosomes by HMC-1 cells decreased when the exosome-cell mixture was incubated on ice, indicating a metabolically active uptake of exosomes (data not shown). In addition, confocal microscopy showed that the exosomal membrane staining is evident intracellularly in the recipient cells (Fig. 6b), which is in accordance with the uptake pattern of exosomes in glioblastoma cells (21).

Interestingly, uptake of PKH67-labelled HMC-1 exosomes were found also by CD34^+^ cells (Fig. 5c, n=4). After 20 hours, 94 ± 4% of the CD34^+^ cells were positive for PKH67, confirming the mRNA and microRNA pathway analysis where the top canonical pathway was cellular development. This shows that exosomes from one cellular source can be taken up by a different type of cell, which in itself increases the complexity by which cells may communicate in the microenvironment.

### HMC-1 exosomes shuttle RNA between cells

Since exosomes were shown to be taken up by HMC-1 and CD34^+^ hematopoietic progenitor cells, we aimed to examine whether these vesicles are capable of delivering their RNA content to the recipient cells. To determine this, exosomal RNA was labelled by culturing HMC-1 donor cells in the presence of radioactive uridine. Exosomes were then isolated from these donor cells and co-cultured with HMC-1 and CD34 recipient cells in separate experiments. The radioactive signal of the RNA isolated from the recipient cells was measured by scintillation counts. The result showed that mast cell-derived exosomes transfer RNA to both human mast cells (0 hour: 40 cpm, 20 hours: 392 cpm, n=2) and to CD34 cells (0 hour: 52 cpm, 20 hours: 517 cpm, n=1) (Fig. 6d).

The discovery of the “cellular development, hematological system development and function, hematopoiesis” network by IPA, together with previous findings that exosomal mRNA delivered to recipient cells is functional, argues that the exosomal RNA delivered to CD34^+^ cells is also functional. The exosomal shuttle of genetic material between mast cells and CD34^+^ cells may represent a regulatory signal by which the mast cells send genetic information to other mast cells and to CD34^+^ cells, either in the airway microenvironment or over a distance.

Previous findings, showing that the RNA content of exosomes is altered depending on the condition that they are released under (14), together with this study's findings that the RNA content of exosomes and cells differ, indicate that cells harbour a specific mechanisms for selective packaging of RNA into exosomes and, thus, deliver a specific message. The identification of these packaging mechanisms are beyond the scope of this work, although examination of mechanisms for such packaging system have been started. Interestingly, Batagov et al. have identified a nucleotide pattern enriched in secreted RNAs, which the authors propose targeting the RNA to exosomes (36).

## Conclusions

In this study, we show that human mast cell-derived exosomes contain RNA and that this RNA can be shuttled to other human mast cells and to human CD34^+^ hematopoietic progenitor cells. The exosomal RNA consists of mRNA and microRNA, and the RNA profile differs substantially between exosomes and their donor cells, arguing that at least a portion of the exosomal RNA content is depending on specific packaging mechanisms. These findings suggest that exosomal shuttle of RNA (esRNA) between cells is a powerful mode of cell-to-cell communication in human cells. Lastly, exosomes may provide an efficient vector to deliver specific RNA molecules in gene therapy.

## Methods

### Cell culture and exosome release

The human mast cell line HMC-1 (Dr. Joseph Butterfield, Mayo Clinic, USA) was cultured in IMDM containing 10% depleted fetal bovine serum (FBS), 100 U ml^−1^ penicillin, 100 µg ml^−1^ streptomycin, 2 mM L-glutamine and 1.2 mM alpha-thioglycerol (all from Sigma-Aldrich, St Louis, MO, USA). To eliminate exosomes present in serum, FBS was ultracentrifuged at 120,000 g for 90 minutes using a Ti70 rotor (Beckman optima LE-80k Ultracentrifuge, Brea, CA, USA). Peripheral blood mononuclear cells (PBMC) were prepared from peripheral blood of healthy subjects by Ficoll-Plaque density separation. The CD34^+^ cells were obtained from the PBMC by positive isolation using magnetic separation according to the manufacturer's instruction (Miltenyi Biotec, Germany). CD34^+^ cells were cultured in IMDM containing 10% FBS, 100 U ml^−1^ penicillin, 100 µg ml^−1^ streptomycin, 2 mM L-glutamine and 1 mM sodium pyruvate (all from Sigma-Aldrich). The purity of the separations ranged between 60 and 85%, as analysed by flow cytometry using antibody against CD34 (BD Biosciences, Erembodegem, Belgium) in combination with 7-AAD for viability. Exosomes were isolated according to Valadi et al. (20). Briefly, cells were stimulated with 1 µM calcium ionophore (Sigma-Aldrich) for 30 minutes, centrifuged at 500 g for 10 minutes followed by 16,500 g for 20 minutes to eliminate cells and cell debris, and finally filtered through 0.22 µm filters. Exosomes were pelleted by ultracentrifugation (Beckman Ti70 rotor) at 120,000 g for 70 minutes. For PKH67 transfer experiments, exosomes were washed once in a large volume PBS. For electron microscopy, the exosome pellet was diluted in a large volume PBS, filtrated through 0.1 µm filters and pelleted by ultracentrifugation. Exosomes were measured for their protein content using BCA™ Protein Assay Kit (Pierce, Thermo Scientific). Cell viability was assessed using trypan blue exclusion or 7-AAD.

### Flow Cytometry of exosomes

For immunoisolation, 4-µm-diameter aldehyde/sulphate latex beads (Interfacial Dynamics) were incubated with purified anti-CD63 antibody (BD Biosciences) under gentle agitation at RT overnight according to manufacturer's recommendation (Interfacial Dynamics). For FACS analysis, 30 µg of HMC-1 exosomes were incubated with 1.5 × 10^5^ anti-CD63 beads in 30 µl PBS at RT for 15 minutes, the volume was made up to 400 µl and the beads were incubated at 4°C overnight under gentle agitation. The reaction was stopped by incubation in 100 mM glycine for 30 minutes. Exosome-coated beads were washed twice, incubated in 1% human serum at 4°C for 15 minutes, washed twice and incubated with PE-conjugated CD9, CD63 or CD81 antibody or isotype control (BD Biosciences), washed and analysed by flow cytometry using a FACS Scan (Becton Dickinson, San Diego CA). Ten thousand events were computed, and data were analyzed using FlowJo Software (Tree Star, Inc, Ashland, OR, USA).

### Electron microscopy

The exosome pellet (2 µg) resuspended in PBS was loaded onto formwar carbon-coated grids (Ted Pella Inc, Redding, CA, USA). Exosomes were fixed in 2% paraformaldehyde for 10 minutes, washed in PBS, postfixed in 2.5% glutaraldehyde, washed in dH_2_O, contrasted in 2% uranyl acetate for 15 min and embedded in a mixture of uranyl acetate (0.8%) and methyl cellulose (0.13%) for 10 minutes. Samples were dried and examined in a LEO 912AB Omega electron microscope (Carl Zeiss NTS, Oberkochen, Germany).

### RNA isolation and detection

RNA was isolated using Trizol^®^ (Invitrogen, Paisley, UK) or RNeasy^®^ mini kit (Qiagen, Hilden, Germany) according to the manufacturer's protocol. For co-purification of microRNA and total RNA, the RNA was extracted using Trizol, followed by the RNeasy mini kit. Cells and exosomes were disrupted and homogenized in Buffer RLT (Qiagen) and 3.5 volumes of 100% ethanol were added to the samples prior to use of the RNeasy mini spin column. The rest of the procedure was performed according to the manufacturer's protocol. Detection and quality control of RNA was performed using capillary electrophoresis and Agilent RNA 6000 Nano Kit according to the manufacturer's protocol (Agilent 2100 Bioanalyzer, Agilent Technologies, Foster City, CA, USA).

### MicroRNA chip analysis and profiling

Identification of microRNA was performed by Exiqon (www.exiqon.com). Briefly, the quality of the total RNA was verified by an Agilent 2100 Bioanalyzer. Total RNA from the exosome and the HMC-1 cell samples were labelled with Hy3™ and Hy5™ fluorescent stain, respectively, using the miRCURY Hy3/Hy5 power labelling kit. The Hy3-labelled exosome samples and a Hy5-labelled mast cells were mixed pair-wise and hybridized to the miRCURY LNA array (v9.2). The hybridization was performed according to the miRCURY LNA array manual using a Tecan HS4800 hybridization station (Tecan Systems, Inc., San Jose, CA, USA). The miRCURY LNA array microarray slides were scanned by a ScanArray 4000 XL scanner (Packard Biochip Technologies, Billerica, MA, USA) and the image analysis was carried out using the ImaGene 6.1.0 software (BioDiscovery, Inc, El Segundo, CA, USA). The quantified signals were normalised using the global Lowess (LOcally WEighted Scatterplot Smoothing) regression algorithm. MicroRNA with signals equal to or below the background signal in 2 or more of the 4 replicate measurements were identified as absent in that slide. The limit for a miRNA to be listed as detectable was set to signal intensities higher than 3 × background (3 × median Hy3 or Hy5 for the total slide). In addition, where signals were detected for <3 of the slides, they were considered unreliable and excluded from sets of detected miRNAs. The experiment was performed in triplicate samples. The signal was calculated as the mean value of the log2MeanRatio Hy3/Hy5 for the triplicates ± SD.

### Affymetrix microarray analysis of exosomes and donor HMC-1 cells

DNA microarray analysis was performed on exosomes and their donor HMC-1 cells (n=4) using the Affymetrix Human Genome U133 Plus 2.0 array, probing for more than 38,500 different genes (HuGe U133 Plus 2.0 GeneChip, Affymetrix, Santa Clara, CA, USA). The experiment was performed by SweGene (http://www.lth.se/index.php?id=7789) according to Affymetrix guidelines and then analysed further by ATLAS Biolabs GmbH (www.atlas-biolabs.com). The array was performed on 4 replicate RNA samples extracted using the Trizol method from exosomes and their donor HMC-1 cells. The expression level signals were scaled in GCOS 1.2 to give a median array intensity of 100. This was done to enable different arrays to be compared. Data were then analysed using the bioconductor statistic package R. Data from each group were normalised separately, and background correction according to MAS5 was applied to each group. The data were filtered for the signal intensities, where each signal value of a group is in the 25% highest intensity values. The data were then filtered for those probe sets that have present calls for all experiments in a group for a gene to be present. For a gene to be “unique,” the data were filtered for present calls for all experiments in a group and absent calls for all experiments in the other group. For details on MAS5, please see the Affymetrix documentation. All data are MIAME compliant and the raw data have been deposited in GEO (accession number: GSE25320).

### Ingenuity Pathway Analysis (IPA)

TargetScanHuman 5.1 was applied to predict mRNA targets for the top 5 annotated microRNAs detected in exosomes (miR-663, miR-503, miR-492, miR-498 and miR-671–5p), which generated a list of 500 mRNA. The data (predicted target mRNA and exosomal mRNA detected by Affymetrix microarray) were analysed through the use of IPA (Ingenuity^®^ Systems version 8.0, www.ingenuity.com). By using this program, functional analysis, networks and canonical pathways were generated. Fisher's exact test was used to assign statistical significance (p<0.05), and each network's score was displayed as −log (p value). Biological functions were also assigned to each network.

### Transfer of exosomes to HMC-1 cells and CD34 cells

Exosomes were labelled with the green fluorescent dye PKH67 (Molecular Probes, Invitrogen) according to manufacturer's protocol. PKH67 stained exosomes were washed 4 times using 300 kDa filters (Sartorius Stedim Biotech GmbH, Goettingen, Germany) to remove excess dye and then mixed with HMC-1 cells or CD34^+^ cells in culture. Cells were harvested after different times (HMC-1: 0, 0.5, 1, 1.5, 2, 3, 4, 5, 6, 8, 10, 12, 24, 48 and 72 hours, CD34: 0 hour and 20 hours), washed 3 times and analysed on a FACS Scan (Becton Dickinson, Mountain View, CA) or a FACS ARIA (Becton Dickinson, San Diego, CA) or visualised in a confocal microscopy (Zeiss LSM 510 META) after staining the nucleus with 7-AAD. CD34 cells were stained with PE-labelled anti-CD34 antibody (BD Biosciences) and 7-AAD before acquisition. As control for non-specific labelling of the cells, PBS was PKH67 stained, washed and added to the cells in a parallel experiment. As control for active uptake, PKH67-stained exosomes and HMC-1 cells were incubated on ice under the same time periods used in parallel experiments. To label RNA in the exosomes, HMC-1 cells were cultured in complete medium supplemented with ^3^H-uridine (Amersham Biosciences AB, Uppsala, Sweden); 1 µl/ml was added 120, 48 and 24 hours before exosome isolation. Exosomes were isolated, washed twice using 10 kDa filters (Amicon Ultra-10, Millipore) and added to HMC-1 cells and CD34 cells in culture. Cells were then harvested and washed twice at 0 hour and 20 hours, and finally RNA was isolated using RNeasy mini kit and the signal of radioactive RNA was measured using scintillation.

### Statistics

The Kruskal Wallis test followed by Mann-Whitney test were used for the statistical analysis, where required, and a p value <0.05 was considered significant. For microarray experiments, please see the respective analysis method.
